# Clinical and economic benefits of irinotecan in combination with 5-fluorouracil and folinic acid as first line treatment of metastatic colorectal cancer

**DOI:** 10.1038/sj.bjc.6600204

**Published:** 2002-06-05

**Authors:** D Cunningham, S Falk, D Jackson

**Affiliations:** Department of Medicine, Royal Marsden NHS Trust, Davis Road, Sutton, Surrey SM2 5PT, UK; Bristol Oncology Centre, Bristol, UK; Aventis Pharma, West Malling, UK

**Keywords:** irinotecan, metastatic colorectal cancer, cost effectiveness analysis, 5-fluorouracil, chemotherapy, first line therapy

## Abstract

The combination of irinotecan plus 5-fluorouracil and folinic acid has clinical and survival benefits over 5-fluorouracil and folinic acid alone in the setting of first line treatment of metastatic colorectal cancer. The aim of this cost-effectiveness analysis was to compare the economic implications, from a UK health commissioner perspective, of the two treatment arms (de Gramont regimen) in this setting. Resource utilisation data collected prospectively during the study were used as a basis for estimating cumulative drug dosage, chemotherapy admistration, and treatment of complications during first line therapy. Resource utilisation associated with further chemotherapy in patients who had progressed during the study was derived from a retrospective case note review. Drug acquisition costs were derived from the British National Formulary (September, 2001) and unit costs for clinical consultation and services were taken from the latest relevant cost database. Cumulative costs per patient associated with further chemotherapy were lower in the irinotecan plus 5-fluorouracil and folinic acid treatment arm. Based on incremental costs per life-year gained of £14 794, the combination of irinotecan plus 5-fluorouracil and folinic acid can be considered cost-effective by commonly accepted criteria compared with 5-fluorouracil and folinic acid alone. Thus, clinical and economic data demonstrate that irinotecan, either in combination with irinotecan plus 5-fluorouracil and folinic acid in the first line setting or as monotherapy in the second line setting, has a major role in the management of metastatic colorectal cancer.

*British Journal of Cancer* (2002) **86**, 1677–1683. doi:10.1038/sj.bjc.6600204 www.bjcancer.com

© 2002 Cancer Research UK

## 

With changes in population demographics, healthcare expenditure is subject to continual review and financial constraint. Clinicians are increasingly being asked to consider both the clinical and economic implications of new treatments and whether they represent value for money compared with currently available options. Irinotecan (Campto®, Aventis Pharma) is now widely accepted in the USA and Europe as an acceptable second line therapy for metastatic colorectal cancer based on clinical and economic considerations (Rougier *et al*, 1998; Iveson *et al*, 1999). Recent data (Douillard *et al*, 2000) have shown that irinotecan, in combination with 5-fluorouracil and folinic acid (5-FU/FA) provides a survival advantage over 5-FU/FA alone, and this indication for first line treatment in metastatic colorectal cancer is reflected in the product licence. The implication of these clinical data have prompted the need for further pharmacoeconomic evaluation in this clinical setting.

Colorectal cancer is the second most common cause of cancer death in the UK and Western developed nations, representing about 12% of all cancer deaths (Schmoll, 1994; Van Triest *et al*, 1995). Annually in the UK, there are over 27 000 new cases of colorectal cancer and over 18 000 deaths due to metastatic disease (Cancer Research Campaign, 1995). In the absence of treatment, median survival from first diagnosis of metastatic colorectal cancer is short (typically 6–9 months) and quality of life is increasingly compromised by both physical and psychological symptoms associated with progression of disease (Seymour *et al*, 1997).

In the UK, palliative chemotherapy is offered to an increasing number of patients with metastatic colorectal disease. At present 5-FU, usually modulated by FA, is regarded as standard first line therapy for metastatic colorectal cancer, with median survival time of about 10–12 months (The Advanced Colorectal Cancer Meta-analysis Project, 1992). Although there is no consensus of a ‘gold standard’ schedule for these drugs, the de Gramont regimen (de Gramont *et al*, 1997) is most commonly used in the UK (Seymour *et al*, 1997). Additionally, in the absence of clear evidence of the best therapeutic option in this setting, other factors such as convenience, cost and quality of life influence clinical practice (Seymour *et al*, 1997).

Irinotecan is a novel chemotherapeutic agent that acts to inactivate DNA topoisomerase 1 and inhibit cell division (Shimada *et al*, 1994). There is no evidence of any cross-resistance with 5-FU (Creemers *et al*, 1994). In the second line setting for metastatic colorectal cancer, irinotecan has been shown to significantly improve survival compared with best supportive care alone (Cunningham *et al*, 1998) or 5-FU with or without FA (Rougier *et al*, 1998). In the first line setting for metastatic disease, Phase II studies in chemotherpy-naïve patients have shown promising activity, with response rates ranging from 19–32% when administered as a single agent (Pitot *et al*, 1994; Conti *et al*, 1996; Rougier *et al*, 1997). These preliminary data were confirmed by a multicentre, randomised, controlled, open-label study (Douillard *et al*, 2000) (see below), and resulted in the combination or irinotecan with 5-FU/FA being licensed as a first line therapy for metastatic colorectal cancer.

Douillard *et al* (2000) compared treatment with the combination of irinotecan+5-FU/FA with 5-FU/FA alone in patients with metastatic colorectal cancer; 385 patients received at least one cycle of treatment; 199 patients received the combination of irinotecan+5-FU/FA (treatment arm A) and 186 patients received 5-FU/FA alone (treatment arm B) (see Table 1

**Table 1:**
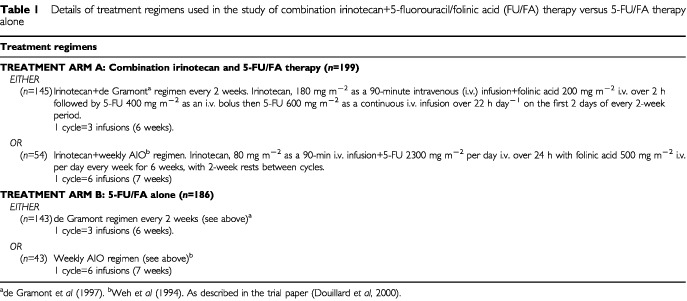
Details of treatment regimens used in the study of combination irinotecan+5-fluorouracil/folinic acid (FU/FA) therapy versus 5-FU/FA therapy alone

for treatment regimens) until the occurrence of disease progression, unacceptable toxicity or withdrawal of consent. In a separate follow-up study in French and UK centres, 62 patients who progressed during the study were followed for up to 3 years until death or trial cut-off date (median (range): 14.7 (11.5–21.1) months).

The aim of this study was to compare the economic implications, from a UK health commissioner perspective, of differences in clinical benefit (response and time to progression) and survival between the combination of irinotecan+5-FU/FA and 5-FU/FA alone as first line therapy for metastatic colorectal cancer. The analysis is based on clinical and resource utilisation data collected prospectively as part of the study (Douillard *et al*, 2000), as well as data relating to further chemotherapy in patients with disease progression during the study which were collected via a retrospective case note review. Costs associated with drug acquisition, treatment delivery, disease complications, and the use of second line chemotherapy were included. Indirect costs, although important, have not been included, as the data have been analysed from the viewpoint of commissioners in the National Health Service (NHS).

## MATERIALS AND METHODS

Clinical and resource utilisation data used for economic assessments in this study are described below. These data provide the basis for calculating the direct costs associated with each treatment arm and for carrying out an economic evaluation comparing the treatment arms with respect to their outcomes and associated costs. Only data relating to patients who received the de Gramont regimen were included in the costs analysis (the AIO regimen is not used in the UK) (Seymour *et al*, 1997), although overall survival was based on all patient data (see Douillard *et al*, 2000). The control arm (5-FU/FA) was valid from a UK perspective as this regimen is most commonly used in the UK (Seymour *et al*, 1997).

All resource utilisation data were collected thoroughly by prospective completion of Case Report Forms in the main trial (Douillard *et al*, 2000) and retrospective collation of data in the follow-up study. Both the clinical endpoints and conclusions drawn from the subset for resource utilisation are valid due to the methods used for collection of data.

### Clinical data

Patient characteristics of the two treatment arms of the study have been previously summarised and reported (see Douillard *et al*, 2000). Treatment with the combination of irinotecan+5-FU/FA was significantly superior to 5-FU/FA alone with respect to response rate (41 *vs* 23%, *P*<0.001) in the evaluable patient population, and was significantly superior to 5-FU/FA alone with respect to median time to disease progression (6.7 months *vs* 4.4 months, *P*<0.001) and median overall survival (16.8 months *vs* 14.0 months, *P*<0.028) in the intent-to-treat patient population. The median survival gain of the combination of irinotecan and 5-FU/FA over 5-FU/FA alone (2.8 months or 0.23 life-years saved) was achieved despite the fact that, of patients who received 5-FU/FA alone, 58.3% received further chemotherapy and 31% were subsequently treated with a regimen containing irinotecan. Life-years saved was a major efficacy parameter used in the most-effectiveness analysis.

### Resource utilisation data

In estimating the economic impact of irinotecan in combination with 5-FU/FA as first line treatment for metastatic colorectal cancer within the UK, it is insufficient to examine only the drug acquisition costs. Hospitalisation (inpatient and outpatient settings) for administration of treatment, nursing time and equipment use must also be considered. In addition, costs associated with the toxicity of treatment, most commonly diarrhoea and neutropenia, and complications of the disease need to be examined. These later costs can be broadly categorised as hospitalisation costs (other than for routine administration of chemotherapy), consultation costs and costs for clinical and diagnostic services. At each assessment in the study, any hospital admission since the last visit was recorded, together with the reason for admission, type of ward and length of stay. General practitioner (GP) consultations and nurse visits, as well as inpatient and outpatient consultations with clinical personnel, were also documented. These data were recorded prospectively during the study as an integral part of the Case Report Form (CRF) and are presented as average figures for the irinotecan+5-FU/FA treatment arm and the 5-FU/FA treatment arm.

Consideration of resource utilisation associated with further chemotherapy following disease progression was essential in order to present a balanced representation of the costs of treating patients in the first line setting. Over the trial and follow-up period, 39.4% of patients randomised to treatment with the combination of irinotecan+5-FU/FA and 58.3% of patients randomised to treatment with 5-FU/FA alone failed first line therapy, i.e., progressed during the study and received further chemotherapy. In most patients, these data relate to second line therapy, although it is recognised that a small proportion may also relate to third line therapy. Data relating to resource utilisation associated with progression and second line therapy were collected via a retrospective case note review.

### Drug acquisition costs during the study

All costs of changes were collected until progression or end of this study. The cumulative dose per patient (mg) was derived from the trial data (Douillard *et al*, 2000) using a mean body surface area of 1.8 m^2^ collected in the trial. The median treatment duration was shorter in the de Gramont 5-FU/FA treatment arm than in the irinotecan+5-FU/FA combination treatment arm: 18.0 *vs* 24.6 weeks, respectively) (Douillard *et al*, 2000). Correspondingly, the calculated cumulative number of infusions per patient given over the study period was lower with 5-FU/FA than with the combination treatment (9.66 *vs* 12.08 infusions, respectively) (Tables 1 and 2

**Table 2:**
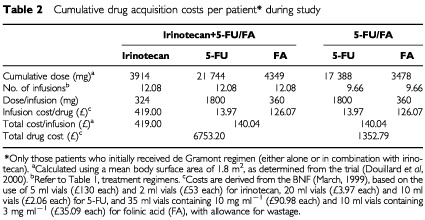
Cumulative drug acquisition costs per patient* during study

). It is useful to note at this point that the higher treatment duration with irnotecan+5-FU/FA combination arm is reflective of the safety of the regimen. Thus whilst more cycles may appear to constitute higher costs, these need to be offset against the gains provied in terms of increased survival.

Cumulative drug costs per patient were based on costs given in the British National Formulary (BNF; September 2001) with allowance for wastage. Where there was more than one option for the same product, the lowest cost alternative was used for specific vial sizes. Drug costs were calcualted by estimating the number of vials needed to provide the required dose for each infusion and then multiplying by the mean number of infusions per patient of each treatment.

### Treatment administration costs during the study

As administration of the study treatment was defined in the protocol, data relating to treatment delivery were not collected on the CRF. Both treatment arms required insertion of a tunnelled central line catheter by a doctor as well as the use of an infusional device. Prospective data collection provided an estimate of the proportion of inpatient hospitalisations and day hospital attendances required per infusion in each treatment arm (see Table 3

**Table 3:**
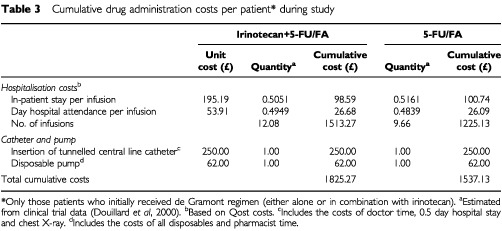
Cumulative drug administration costs per patient* during study

).

Hospitalisations were costed on the basis of 1997/98 extra-contractual referral tariffs (i.e., tariffs negotiated between a hospital and a local authority other than that responsible for the hospital) collected from 12 NHS Trusts in the UK (Qost database). General medicine and surgery ward tariffs were divided by the official average length of stay published by the Department of Health (1993/94) (Department of Health Government Statistical Service, 1993) in order to obtain a ‘*per diem*’ cost. The tariffs covered all types of inpatient resources consumed.

### Costs associated with complications of treatment and disease during the study

All unplanned hospitalisations were recorded prospectively on the Case Report Form. Hospital admissions due to complications included those associated with adverse events resulting from administration of chemotherapy and those resulting from disease complications. Data for hospitalisation due to planned chemotherapy administration were excluded. However, if hospitalisation for chemotherapy administration was prolonged because of toxicity, the hospital stay was retained in the calculation. Outpatient visits were also categorised by the type of consultation. Other resource items recorded on the CRFs related to the number of nurse visits and radiotherapy (Table 4

**Table 4:**
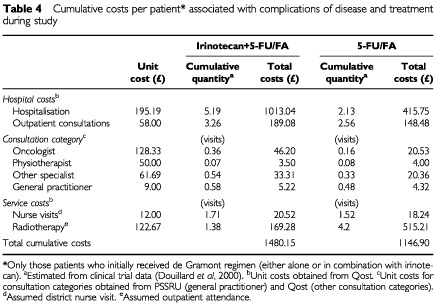
Cumulative costs per patient* associated with complications of disease and treatment during study

).

Unit costs for hospitalisation, specialist consultations and diagnostic costs were derived from the Qost database (1997/98) as previously described. The consultation tariff included the costs of procedures performed during the attendance. As diagnostic tests were usually performed at hospital, outpatient Trust tariffs were used in the costing of these services. Health professional, nurse and GP consultations were costed on the basis of Personal Social Services Research Unit (PSSRU) handbook (Netten and Dennett, 1998). As nurse and health professionals costs were given in hours, it was assumed that each consultation would be of 0.5 h duration.

Overall cumualtive costs per patient associated with complications in each treatment arm were calculated using estimates of the cumulative number of hospitalisations, consultations, and clinical and diagnostic services required per treatment arm derived from the trial data.

### Cost associated with further chemotherapy

Data relating to resource utilisation associated with progression and further chemotherapy were derived from a retrospective case note review for 62 patients in centres in France and the UK who had progressed during the study. Costs associated with further therapy following disease progression were categorised as either drug costs (i.e., drug acquisition costs and costs associated with treatment delivery) or disease progression costs (i.e., costs associated with further hospitalisation and radiotherapy).

Information was collected on all treatment regimens used for futher chemotherapy in each treatment arm. Wherever possible, calculation of the number of infusions administered for each treatment regimen was based on published sources (see Table 5

**Table 5:**
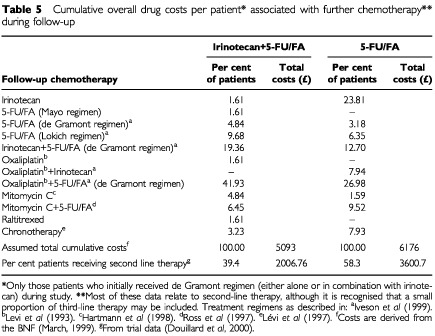
Cumulative overall drug costs per patient* associated with further chemotherapy** during follow-up

). Assumed cumulative costs per patient for each treatment regimen were based on acquisition costs given in the British Natural Formulatory (BNF, March 1999), with allowance for wastage, and included costs associated with treatment delivery. The actual cumulative drug costs per patient associated with further chemotherapy were then calculated by multiplying the total assumed cumulative drug costs per patient of futher therapy by the proportion of patients in each treatment arm who had received further therapy during follow-up (i.e. 39.4% in the irinotecan+5-FU/FA combination treatment arm and 58.3% in the 5-FU/FA treatment arm) (Douillard *et al*, 2000).

Retrospective data collection was used to estimate the total cumulative hospitalisation and radiotherapy costs per patient associated with disease progression. The actual cumulative costs per patient associated with disease progression were then calculated by multiplying by the proportion of patients in each treatment arm who had progressed during the study.

### Overall costs

In the setting of first line therapy, the overall cumulative costs for each treatment arm represented the total sum of costs associated with first line therapy and costs associated with disease progression, including further chemotherapy. The incremental costs and outcome with each treatment arm were compared. A cost effectiveness ratio per life year gained (LYG) was calculated as the difference in overall costs between the combination of irinotecan+5-FU/FA and 5-FU/FA alone divided by the difference in median survival between the combination regimen and 5-FU/FA therapy alone (i.e., 2.8 months or 0.23 life-years saved; see Douillard *et al*, 2000). A sensitivity analysis, based on UK data alone to reflect the local situation, was also performed (Table 6

**Table 6:**
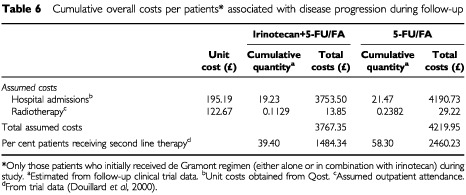
Cumulative overall costs per patients* associated with disease progression during follow-up

).

## RESULTS

### Costs during the study

Cumulative costs during the study are summarised by treatment arm in Tables 2, 3 and 4. As anticipated, cumulative drug acquisition costs during the study were substantially higher in the irinotecan+5-FU/FA combination treatment arm than the 5-FU/FA treatment arm (£6753 *vs* £1353, respectively) (Table 2). Cumulative costs associated with treatment delivery (Table 3) and with drug toxicity and disease complications (Table 4) were generally similar in each treatment arm.

### Costs during follow-up

However, the higher cumulative costs per patient associated with first line treament with irinotecan+5-FU/FA were offset by substantially lower cumulative costs per patient during the trial and follow-up period, probably attributable to reduced disease progression in the combination treatment arm (39.4% *vs* 58.3% of patients, respectively) (Douillard *et al*, 2000). Both cumulative drug costs per patient (£2007 in the irinotecan+5-FU/FA arm *vs* £3601 in the 5-FU/FA arm) (Table 5), and cumulative diseaase progression costs per patient during follow-up (£1484 *vs* £2460, respectively) (Table 6) were up to 45% lower in the combination treatment arm.

### Total costs

When cumulative costs per patient associated with first line (during study) and further (during follow-up) chemotherapy were considered together, it is apparent that the overall cumulative costs per patient in the irinotecan+5-FU/FA combination treatment arm were higher than those associated with 5-FU/FA alone (difference in costs, £3452) (Table 7

**Table 7:**
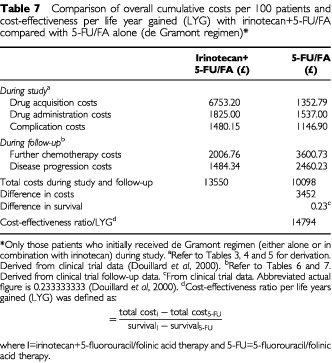
Comparison of overall cumulative costs per 100 patients and cost-effectiveness per life year gained (LYG) with irinotecan+5-FU/FA compared with 5-FU/FA alone (de Gramont regimen)*

).

However, treatment with the combination of irinotecan+5-FU/FA in the first line setting also resulted in a significant gain in median survival over 5-FU/FA alone (0.23 life-years (Douillard *et al*, 2000)). Cost-effectiveness analysis of incremental costs and survival relative to 5-FU, demonstrated that treatment with the combination of irinotecan+5-FU/FA in the first line setting resulted in incremental costs per LYG of £14 794. Sensitivity analyses based on UK data alone showed that there was little change in incremental costs per LYG when rates were varied to reflect UK practice (£16 015).

## DISCUSSION

Combination treatment with irinotecan+5-FU/FA is now licensed as a first line therapy for metastatic colorectal cancer. Compared with 5-FU alone, the combination of irinotecan+5-FU/FA offers a significant survival advantage without detriment to quality of life (Douillard *et al*, 2000).

However, the introduction of new treatments to hospital formularies requires demonstration of cost as well as clinical advantages. The new treatment must be shown to be cost-effective relative to current best practice, specifically with respect to value parameters such as survival gain. Defining arbitrary financial limits based on such value parameters is difficult, particularly as there is a general paucity of guidelines on which to base judgements. Yet choices are inevitable and necessary.

In the UK, there are no guidelines for using clinical and economic evaluations, and no defined limit at which the incremental costs and clinical benefits of a treatment favour its introduction into routine clinical practice. However, tentative limits can be surmised from a recent Department of Health review of available cost-effectiveness studies (Department of Health, 1994). These data (Department of Health, 1994) suggest that incremental costs per LYG of £15 000–£20 000 for a cancer treatment can be considered reasonable, and hence the treatment may be viewed as cost-effective compared with currently accepted best practice. Based on these considerations, treatment with irinotecan+5-FU/FA was associated with only a modest increase in cost compared with 5-FU/FA alone (as supported by sensitivity analyses involving only UK data), which together with the significant survival gain demonstrated for the combination treatment (Douillard *et al*, 2000), justifies its use as a first line therapy for metastatic colorectal cancer.

In conclusion, the results of the cost effectiveness analysis presented in this study, together with clinical evidence (Douillard *et al*, 2000), strongly support the use of irinotecan+5-FU/FA in the setting of first line therapy of metastatic colorectal cancer. Moreover, clinical (Rougier *et al*, 1998) and economic (Iveson *et al*, 1999) studies have confirmed the superiority of irinotecan in the setting of second line therapy. Thus, irinotecan, either alone or in combination with 5-FU/FA, represents an important therapeutic advance in the management of metastatic colorectal cancer.
